# Effect of neonatal reticulocytosis on glucose 6-phosphate dehydrogenase (G6PD) activity and G6PD deficiency detection: a cross-sectional study

**DOI:** 10.1186/s12887-022-03740-1

**Published:** 2022-11-23

**Authors:** Thanaporn Pimpakan, Punchalee Mungkalasut, Pornchinee Tansakul, Makamas Chanda, Watcharapong Jugnam-Ang, Supamas Charucharana, Poonlarp Cheepsunthorn, Suthat Fucharoen, Santi Punnahitananda, Chalisa Louicharoen Cheepsunthorn

**Affiliations:** 1 Medical Sciences Program, Faculty of Medicine, Chulalongkorn University, Bangkok, Thailand; 2 Interdisciplinary Program of Biomedical Sciences, Graduate School, Chulalongkorn University, Bangkok, Thailand; 3 Medical Biochemistry Program, Department of Biochemistry, Faculty of Medicine, Chulalongkorn University, Bangkok, Thailand; 4 Department of Applied Thai Traditional Medicine, Faculty of Science and Technology, Phranakhon Rajabhat University, Bangkok, Thailand; 5 Department of Anatomy, Faculty of Medicine, Chulalongkorn University, Bangkok, Thailand; 6 Thalassemia Research Center, Institute of Molecular Biosciences, Mahidol University, Nakorn Pathom, Thailand; 7 Division of Neonatology, Department of Pediatrics, Faculty of Medicine, Chulalongkorn University, Bangkok, Thailand; 8 Department of Biochemistry, Faculty of Medicine, Chulalongkorn University, 1873 Rama 4 Rd, Pathumwan, Bangkok, 10330 Thailand

**Keywords:** G6PD deficiency, Reticulocyte, Quantitative method, Automated UV enzymatic method

## Abstract

**Background:**

Screening for G6PD deficiency in newborns can help prevent severe hemolysis, hyperbilirubinemia, and bilirubin encephalopathy, as recommended by the World Health Organization (WHO). It has been speculated that the presence of a high number of reticulocytes in newborns interferes with the diagnosis of G6PD deficiency since reticulocytes contain higher amounts of G6PD enzyme than mature erythrocytes. Therefore, the purposes of this study were to assess the effect of reticulocytosis in the determination of blood G6PD activity in Thai newborns by using a novel automated UV-based enzymatic assay and to validate the performance of this assay for the detection of G6PD deficiency in newborn samples.

**Methods:**

The levels of reticulocytes and G6PD activity were measured in blood samples collected from 1,015 newborns. *G6PD* mutations were identified using *Taq*Man^®^ SNP genotyping assay, PCR–restriction fragment length polymorphism (PCR–RFLP), and direct sequencing. The correlation between the levels of reticulocytes and G6PD activity was examined. The performance of the automated method was compared with that of the fluorescent spot test (FST) and the standard quantitative assay.

**Results:**

The automated assay detected G6PD deficiency in 6.5% of the total newborn subjects compared to 5.3% and 6.1% by the FST and the standard method, respectively. The minor allele frequencies (MAFs) of *G6PD Viangchan*^*G871A*^, *G6PD Mahidol*^*G487A*^, and *G6PD Union*^*C1360T*^ were 0.066, 0.005, and 0.005, respectively. The reticulocyte counts in newborns with G6PD deficiency were significantly higher than those in normal male newborns (*p* < 0.001). Compared with normal newborns after controlling for thalassemias and hemoglobinopathies, G6PD-deficient patients with the *G6PD Viangchan*^*G871A*^ mutation exhibited elevated reticulocyte counts (5.82 ± 1.73%, *p* < 0.001). In a group of G6PD normal newborns, the percentage of reticulocytes was positively correlated with G6PD activity (*r* = 0.327, *p* < 0.001). However, there was no correlation between G6PD activity and the levels of reticulocytes in subjects with G6PD deficiency (*r* = -0.019, *p* = 0.881). The level of agreement in the detection of G6PD deficiency was 0.999, while the area under the receiver operating characteristic (AUC) curve demonstrated that the automated method had 98.4% sensitivity, 99.5% specificity, 92.4% positive predictive value (PPV), 99.9% negative predictive value (NPV), and 99.4% accuracy.

**Conclusions:**

We report that reticulocytosis does not have a statistically significant effect on the detection of G6PD deficiency in newborns by both qualitative and quantitative methods.

## Background

Glucose 6-phosphate dehydrogenase (G6PD) deficiency is the most common hereditary blood disorder, affecting millions of people worldwide [1]. G6PD is a rate-limiting enzyme in the hexose monophosphate shunt (HMP) generating reduced nicotinamide adenine dinucleotide phosphate (NADPH), a coenzyme in glutathione metabolism, to defend against oxidative stress in red blood cells [2]. Patients with defective G6PD enzymes are generally asymptomatic unless they are exposed to infection or stimulants, such as fava beans and the antimalarial drug primaquine [1, 3, 4]. G6PD deficiency manifests as acute hemolytic anemia (AHA), hemoglobinuria and favism [1]. In Asians, severe cases in newborns with delayed diagnosis and treatment can develop cerebral jaundice and hyperbilirubinemia, resulting in life-threatening bilirubin neurotoxicity, kernicterus, and mental retardation [3–5]. In Thailand, the prevalence of G6PD deficiency in male newborns is 10.7–17.0% and that of female newborns is 1.2–5.8% [6–8]. Approximately 65% of severe neonatal jaundice and 21.2–22.1% of hyperbilirubinemia were coincidently observed in G6PD-deficient Thai newborns [6, 9]. Thus, the World Health Organization (WHO) has recommended routine screening for G6PD deficiency in newborns living in areas where the prevalence of G6PD deficiency is as high as 3–5% in males to avoid unwanted consequences [10].

G6PD deficiency is an X-linked recessive inborn error commonly observed in hemizygous males, whereas normal, intermediate, or grossly deficient phenotypes depending on a mosaicism of lyonization can be observed in heterozygous females [8]. Heterozygous females with partial G6PD activity may be misdiagnosed. A quantitative G6PD enzymatic activity assay based on a spectrophotometric technique is the reference assay to detect G6PD deficiency and G6PD intermediates [11]. It was suggested by previous studies that the cut-off values for G6PD deficiency and intermediate G6PD should be activity values less than 30% and 30% to 60% of the adjusted male median (AMM), respectively [11–14]. The WHO has recommended that activity values less than 30% and 30%-80% of the normal median should be used instead [15]. Nevertheless, the differences in mosaicism, study techniques, and the reticulocyte/erythrocyte ratio may affect the interpretation of G6PD deficiency [5, 16, 17]. In particular, high levels of G6PD-rich reticulocytes, known as reticulocytosis, commonly observed in newborns may be an important interfering factor for the detection of G6PD deficiency in newborns, leading to false-negative results [18, 19].

Therefore, the present study aimed to investigate the influence of reticulocytes on newborn G6PD activity determined by different methods, including the fluorescent spot test (FST) and two quantitative assays (standard and automated UV enzymatic methods). The performance of the automated method was compared with that of FST and the standard quantitative method.

## Methods

### Subjects and sample collection

Approval of Research Ethics Committee, Faculty of Medicine, Chulalongkorn University (COA No. 823/2020, IRB No. 254/62) was obtained for this study. A total of 1,015 newborns (gestation ≥ 37 weeks, birth weight ≥ 2,000 g.), encountered at King Memorial Chulalongkorn Hospital, Bangkok, Thailand, from February to August 2021, were enrolled in the study. EDTA blood samples of newborn infants were collected on the 3^rd^ day of life during a routine screening for phenylketonuria (PKU) and congenital hypothyroidism and immediately screened for G6PD deficiency. Reticulocyte counts and hemoglobin (Hb) levels were measured using an autohematology analyzer BC-6200 (Mindray Medical International, China).

### Fluorescent spot test (FST)

The FST (R&D Diagnostic, Athens, Greece) was performed according to the manufacturer’s instructions. Briefly, a mixture of blood sample (1.25 µl) and the reaction reagents (25 µl) was incubated at 37 °C for 15 min, spotted on filter paper, and allowed to air-dry in the dark at room temperature for 15 min. Then, spotted samples were exposed to UV light to visualize the emitted fluorescence of the reaction. Samples with moderate to strong fluorescence were categorized as G6PD normal, whereas nonfluorescent samples were categorized as G6PD deficient.

### Quantitative G6PD spectrophotometric assay

The quantitative G6PD assay kit (Cat No. G7583; Pointe Scientific INC., Canton, Michigan, USA) was used as the reference assay for the measurement of G6PD activity in this study. All whole blood samples were analyzed following the manufacturer’s instructions. Normal and deficient G6PD controls (G6888, G5888; Trinity Biotech, Bray, Co. Wicklow, Ireland) were included in each assay run. The absorbance was measured at 340 nm over a period of 5 min at 37 °C in a temperature-regulated spectrophotometer (Shimadzu UV-1800, Shimadzu, Japan). The results from each assay run were considered valid when the activity of G6PD controls was within the reference range. Hemoglobin (Hb) in each blood sample was measured using an auto hematology analyzer BC-6200 (Mindray Medical International, China). G6PD activity was calculated from the change in absorbance over a 5-min period and presented as U/g Hb.

### Automated UV enzymatic assay

In brief, 20 μl of the hemolysate prepared from packed red blood cells was added to 1 ml of distilled water and mixed for 5 min. G6PD activity was then measured at 37 °C according to a previously described protocol using a clinical chemistry analyzer (BS-360E, Mindray Medical International, China) [11]. The rate of absorbance change (∆A/min) was calculated by (∆A/min-sample)-((∆A/min blank) and normalized to Hb (g/dL) according to the manufacturer’s instructions. Sample G6PD activity was considered valid if the control G6PD activity fell within the reference range.

### Identification of *G6PD* mutations

Genomic DNA was extracted from blood samples using a Nucleospin® Blood kit (MACHEREY–NAGEL GmbH & Co. KG, Düren, Germany) according to the manufacturer’s recommendations. A *Taq*Man^®^ SNP genotyping assay (Applied Biosystem, Foster City, CA, USA) was performed to screen for *G6PD Mahidol*^*G487A*^ and *G6PD Viangchan*^*G871A*^. Amplification, real-time detection, and result analysis were performed on a StepOnePlus Real-time PCR (Applied Biosystem, Foster City, CA, USA) following the manufacturer’s instructions. PCR–RFLP was performed to detect *G6PD Canton*^*G1376T*^*, G6PD Union*^*C1360T*^, and *G6PD Kaiping*^*G1388A*^ in G6PD deficient and intermediate samples as described previously [20]. For G6PD-deficient patients with unknown mutations, the coding exons of G6PD (exons 3–12) were amplified using primers published previously [21]. The PCR products were sequenced (Macrogen, Korea). BioEdit software version 2.1 was used to identify the mutations using the *G6PD* reference sequence (NC 000,023.11) from NCBI.

### Statistical analysis

The distribution of data was assessed using Kolmogorov–Smirnov/Shapiro–Wilk tests. The parametric tests were performed in normally distributed data. G6PD activity values of male and female newborns are expressed as the median ± interquartile range (IQR). The cut-off values of G6PD activity were defined as follows: deficiency < 30% and intermediate 30%—80% of the normal median [14]. Subjects with G6PD activity > 80% of the normal median were considered normal [12]. Linear regression was performed to evaluate the correlation between G6PD activity from two G6PD enzymatic assays and between reticulocytes and G6PD activity. Cohen’s Kappa statistic was performed to evaluate the degree of agreement between the two G6PD enzymatic assays. The area under the receiver operating characteristic (ROC) curve (AUC) was calculated to test the efficacy of the automated UV enzymatic assay in the detection of G6PD deficiency and intermediates. Finally, the performance of the automated UV enzymatic assay in terms of sensitivity, specificity, positive predictive values (PPVs), negative predictive values (NPVs), and accuracy was evaluated. The two-tailed Student’s *t*-test was used to analyze the differences in quantitative variables. All statistical analyses were conducted in SPSS software version 22 (IBM Corp., Armonk, N.Y., USA).

## Results

A total of 1,015 Thai newborns consisted of 502 males and 513 females. The demographic data of the newborns are summarized in Table 1. The mean gestational age and postpartum age of all subjects were 39.2 ± 18.0 weeks and 2.7 ± 0.8 days, respectively. The mean birth weight of male newborns was 3,179.5 ± 414.1 g, which was significantly higher than that of female newborns (3,061.4 ± 389.4 g; *p* < 0.001) (Table 1).

**Table 1:** Demographic data of newborn subjects

	**Total (*n* = 1,015)**	**Male** **(*n* = 502)**	**Female (*n* = 513)**	*p*-value*
Gestational age (wk.)	39.2 ± 18.0	38.3 ± 1.0	40.1 ± 25.3	0.152
Postpartum age (day)	2.7 ± 0.8	2.8 ± 0.8	2.7 ± 0.8	0.191
Birth weight (g)	3,119.8 ± 405.9	3,179.5 ± 414.1	3,061.4 ± 389.4	< 0.001
G6PD deficiency by FST (n) (%)	54 (5.3)	46 (9.2)	8 (1.6)	< 0.001
G6PD deficiency by spectrophotometric assay (n) (%)	62 (6.1)	50 (10.0)	12 (2.3)	< 0.001
G6PD deficiency by automated UV enzymatic assay (n) (%)	66 (6.5)	52 (10.4)	14 (2.7)	< 0.001

Values are expressed as the mean ± SD

^*^ Comparison between males and females

The aetiology of haemolytic disease of the foetus and newborn (HDFN) can be either immune or non-immune mediated. Blood group incompatibility between the mother and the fetus is the main cause of immune-mediated HDFN. The causes of nonimmune-mediated HDFN include enzyme abnormalities, RBC membrane problems, thalassemia, and hemoglobinopathies. In this study population, we found 8 newborns with ABO incompatibility, 44 newborns with thalassemia/hemoglobinopathies, and 1 coincidence of ABO incompatibility and thalassemia/hemoglobinopathies. The incidence of G6PD deficiency in the male and female populations of this study using three diagnostic techniques is shown in Table 1. The spectrophotometric assay was used as the gold standard method to verify the results of the other two techniques.

**Fig. 1:**
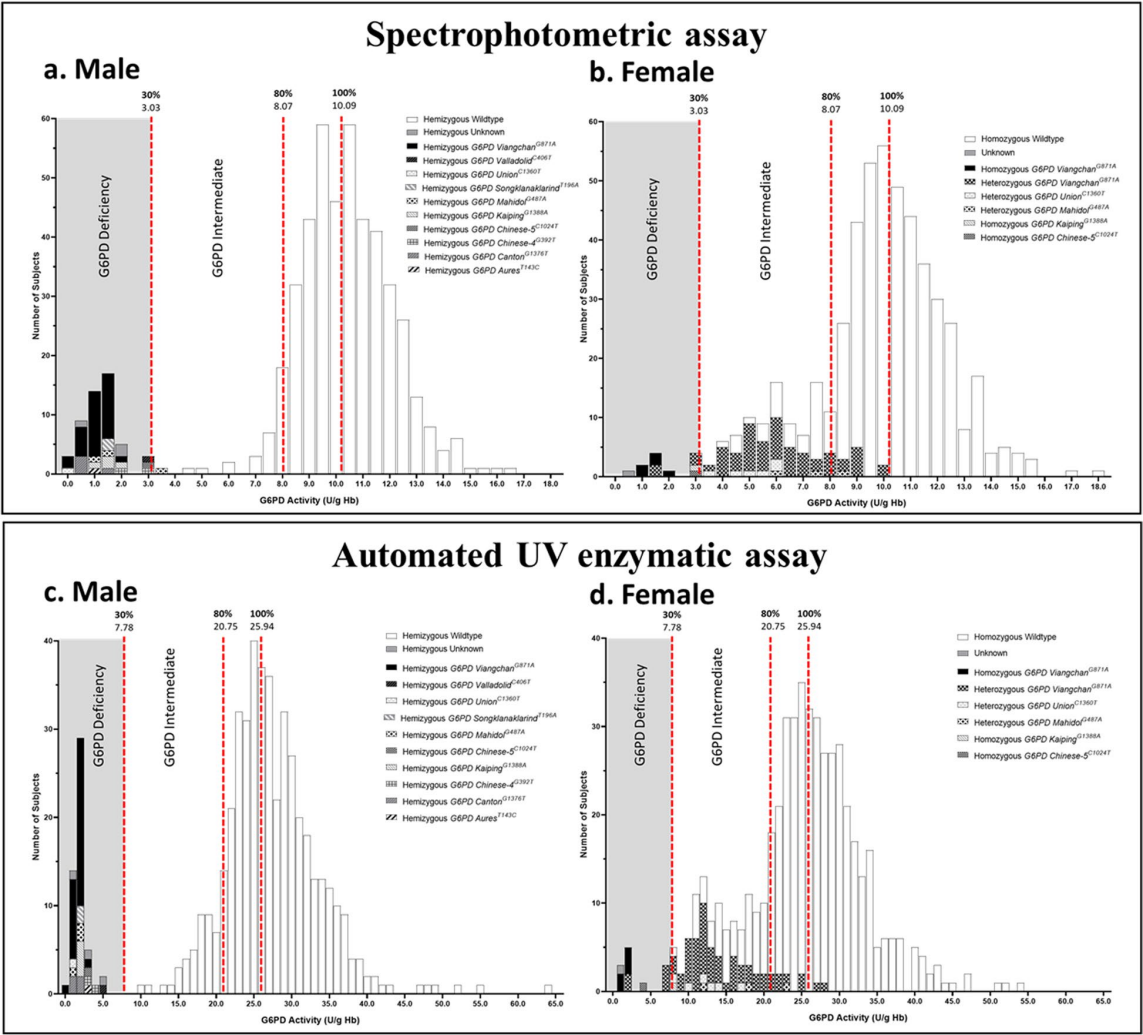
The histogram represents the correlation between *G6PD* mutations and G6PD activities in the study population. The G6PD activities in (**a, c)** males and (**b, d)** females as measured by (**a, b**) the spectrophotometric and (**c, d**) automated UV enzymatic assays

After the Kolmogorov–Smirnov and Shapiro–Wilk tests for normality, G6PD activity of neonatal males as measured by spectrophotometric and automated UV enzymatic assays showed a bimodal distribution (*p* < 0.001) and that of neonatal females showed a trimodal distribution of homozygous and heterozygous G6PD deficient and homozygous normal samples (*p* < 0.001) (Fig.1a-d). The median ± IQR of G6PD activity of males (10.1 ± 2.5 U/g Hb) and females (10.0 ± 2.7 U/g Hb) obtained from the spectrophotometric assay were not significantly different (*p* = 0.848 by the Mann–Whitney test). The results from the automated UV enzymatic method demonstrated that there was no significant difference in median G6PD activity between males (25.9 ± 7.8 U/g Hb) and females (25.6 ± 9.0 U/g Hb) (*p* = 0.375 by Mann–Whitney test). The G6PD reference values in newborn subjects determined by spectrophotometric assay were as follows: deficiency < 3.03 U/g Hb, intermediate 3.03—8.07 U/g Hb, and normal > 8.07 U/g Hb, whereas that determined by the automated UV enzymatic assay was as follows: deficiency < 7.78 U/g Hb, intermediate 7.78—20.75 U/g Hb, and normal > 20.75 U/g Hb (Fig. 1a-d and Table 2). The median values of G6PD activity of deficiency, intermediate, and normal males and females obtained from both assays are shown in Table 3.

**Table 2:** Proportion of newborn subjects with G6PD status determined by the spectrophotometric and automated UV enzymatic assays

		***Spectrophotometric assay (n)***			
		**Deficiency**	**Intermediate**	**Normal**	**Total**
		**(< 3.03 U/g Hb)**	**(3.03–8.07 U/g Hb)**	**(> 8.07 U/g Hb)**	
***FST***					
**Male**	**Deficiency**	46	-	-	46
	**Normal**	4	29	423	456
	**Total**	50	29	423	502
**Female**	**Deficiency**	8	-	-	8
	**Normal**	4	87	414	505
	**Total**	12	87	414	513
***Automated UV enzymatic assay***					
**Male**	**Deficiency (< 7.78 U/g Hb)**	50	2	0	52
	**Intermediate (7.78—20.75 U/g Hb)**	0	11	36	47
	**Normal (> 20.75 U/g Hb)**	0	16	387	403
	**Total**	50	29	423	502
**Female**	**Deficiency (< 7.78 U/g Hb)**	11	3	0	14
	**Intermediate (7.78—20.75 U/g Hb)**	1	69	41	111
	**Normal (> 20.75 U/g Hb)**	0	15	373	388
	**Total**	12	87	414	513

**Table 3:** G6PD activity and reticulocyte count in the G6PD deficiency, intermediate, and normal newborn groups

Parameter	G6PD Normal			G6PD Intermediate			G6PD Deficiency		
	**Total**	**Male**	**Female**	**Total**	**Male**	**Female**	**Total**	**Male**	**Female**
**Spectrophotometric assay (n)**	**837**	**423**	**414**	**116**	**29**	**87**	**62**	**50**	**12**
**G6PD activity (U/g Hb)**	10.48 ± 2.18	10.52 ± 2.15	10.45 ± 2.23	6.34 ± 2.40	7.63 ± 1.01	6.04 ± 1.92	1.33 ± 0.75	1.21 ± 0.76	1.56 ± 1.67
**Reticulocyte count (%)**	4.78 ± 1.29	4.56 ± 1.20	5.01 ± 1.33	5.03 ± 1.43	4.31 ± 1.69	5.27 ± 1.25	5.82 ± 1.73^a^	5.86 ± 1.90^b^	5.65 ± 0.69^c^
**Automated UV enzymatic assay (n)**	**791**	**403**	**388**	**158**	**47**	**111**	**66**	**52**	**14**
**G6PD activity (U/g Hb)**	27.45 ± 6.63	27.26 ± 6.68	27.50 ± 6.63	16.49 ± 6.37	18.26 ± 2.81	14.75 ± 6.22	1.81 ± 0.90	1.76 ± 0.70	2.11 ± 5.44
**Reticulocyte count (%)**	4.82 ± 1.29	4.60 ± 1.20	5.04 ± 1.34	4.76 ± 1.37	3.94 ± 1.25^b^	5.10 ± 1.28	5.83 ± 1.71^a^	5.91 ± 1.88^b^	5.52 ± 0.75^c^

Values are expressed as the mean ± SD

^a^ compared to the normal group (*p* < 0.001) (*t*-test)

^b^ compared to males in the normal group (*p* < 0.001) (*t*-test)

^c^ compared to females in the normal group (*p* < 0.05) (*t*-test)

### The correlation between G6PD activity and *G6PD* mutations in the study population

Of all 62 patients with G6PD deficiency and 116 patients with intermediate G6PD detected by spectrophotometric assay, 81 patients carried *G6PD Viangchan*^*G871A*^ (30 hemizygous deficient males, 1 hemizygous intermediate male, 5 homozygous deficient females, 4 heterozygous deficient females, 41 heterozygous intermediate females), 5 patients carried *G6PD Mahidol*^*G487A*^ (2 hemizygous deficient males, 1 hemizygous intermediate male, 2 heterozygous intermediate females), 7 patients carried *G6PD Union*^*C1360T*^ (1 hemizygous deficient males and 6 heterozygous intermediate females), 5 patients carried *G6PD Kaiping*^*G1388A*^ (4 hemizygous deficient males and 1 homozygous deficient females), 4 hemizygous deficient males carried *G6PD Canton*^*G1376T*^, 2 patients carried *G6PD Chinese-5*^*C1024T*^ (1 hemizygous intermediate males and 1 homozygous deficient females), 2 hemizygous deficient males carried *G6PD Songklanaklarind*^*T196A*^, 2 hemizygous deficient males carried *G6PD Chinese-4*^*G392T A*^, 1 hemizygous deficient male carried *G6PD Valladolid*^*C406T*^, 1 hemizygous deficient male carried *G6PD Aures*^*T143C*^ (Table 4). In the remaining 4 deficient patients, we did not find mutations in the *G6PD* gene by a direct sequencing method. The minor allele frequencies (MAFs) of *G6PD Viangchan*^*G871A*^, *G6PD Mahidol*^*G487A*^, and *G6PD Union*^*C1360T*^ in the study population were 0.066, 0.005, and 0.005, respectively. The MAFs of the other mutations, including *G6PD Kaiping*^*G1388A*^, *G6PD Canton*^*G1376T*^, *G6PD Songklanaklarind*^*T196A*^, *G6PD Chinese-4*^*G392T*^, *G6PD Chinese-5*^*C1024T*^, *G6PD Aures*^*T143C*^, and *G6PD Valladolid*^*C406T*^*,* were 0.004, 0.003, 0.001, 0.001, 0.002, 0.001, and 0.001, respectively. G6PD activities in each genotype of *G6PD* mutations found in male and female newborns are presented in Table 4.

**Table 4:** G6PD activity and all genotypes of subjects enrolled in the study

Gender	Genotype (n)	Spectrophotometric assay (U/g Hb) (Range)	Automated UV enzymatic assay (U/g Hb) (Range)
**Male**	**Hemizygous *G6PD Viangchan*** ^***G871A***^**(32)**	1.06 ± 0.59 (0.00–2.13)	1.76 ± 0.59 (0.30–2.55)
	**Hemizygous *G6PD Canton*** ^***G1376T***^**(4)**	0.67 ± 0.70 (0.43–1.34)	1.42 ± 0.65 (0.89–1.70)
	**Hemizygous *G6PD Kaiping*** ^***G1388A***^**(4)**	1.58 ± 0.51 (1.21–1.87)	1.87 ± 0.29 (1.69–2.06)
	**Hemizygous *G6PD Mahidol*** ^***G487A***^**(3)**	1.42 (1.07–3.48)	1.64 (1.24–1.70)
	**Hemizygous *G6PD Songklanaklarind*** ^***T196A***^**(2)**	1.59 (1.44–1.74)	2.09 (1.80–2.38)
	**Hemizygous *G6PD Chinese-4*** ^***G392T***^**(2)**	2.44 (1.95–2.92)	3.5 (3.40–3.61)
	**Hemizygous *G6PD Union*** ^***C1360T***^**(1)**	0.16	1.31
	**Hemizygous *G6PD Chinese-5*** ^***C1024T***^**(1)**	3.16	3.24
	***Hemizygous G6PD Valladolid*** ^***C406T***^**(1)**	2.76	5.47
	**Hemizygous *G6PD Aures*** ^***T143C***^**(1)**	1.21	3.36
	**Hemizygous Unknown** **(3)**	1.820 (0.62–1.83)	2.92 (0.89–4.63)
**Female**	**Heterozygous *G6PD Viangchan*** ^***G871A***^**(57)**	5.85 ± 2.47 (1.40–10.24)	12.57 ± 7.34 (1.89–28.13)
	**Heterozygous *G6PD Union*** ^***C1360T***^**(6)**	5.23 ± 1.58 (3.45–5.93)	12.23 ± 5.19 (7.88–16.51)
	**Heterozygous *G6PD Mahidol***^***G487A***^**(4)**	7.65 ± 2.57 (5.83–8.40)	18.21 ± 11.60 (11.76–23.36)
	**Homozygous *G6PD Viangchan*** ^***G871A***^**(6)**	1.41 ± 0.60 (0.91–1.83)	1.55 ± 0.59 (1.15–1.99)
	**Homozygous *G6PD Kaiping*** ^***G1388A***^ **(1)**	2.86	7.75
	**Homozygous *G6PD Chinese-5*** ^***C1024T***^**(1)**	2.93	3.7
	**Unknown (1)**	0.47	1.27

### Correlation between G6PD activity and reticulocyte count

**Fig. 2:**
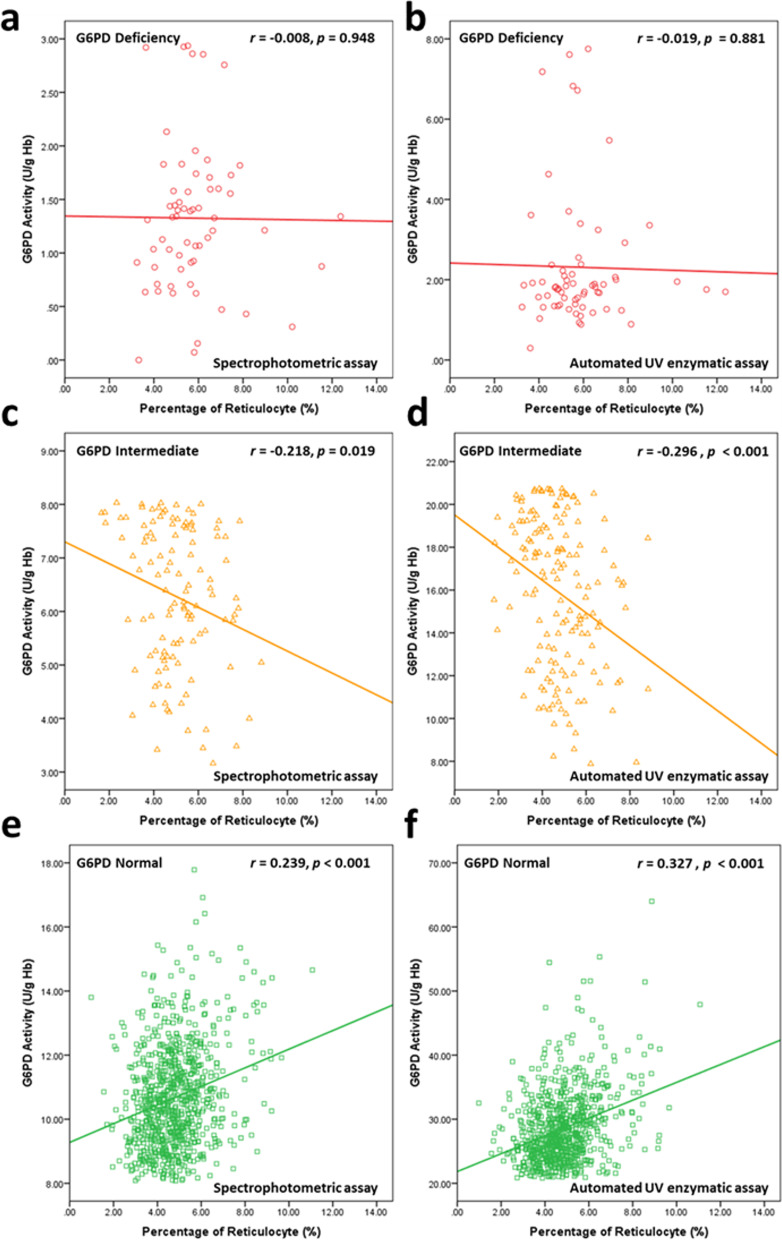
Correlation between G6PD activity and the percentage of reticulocytes in newborns with various G6PD statuses. G6PD activity and reticulocyte counts in newborns with **a**, **b** G6PD deficiency, **c**, **d** G6PD intermediate, and **e**, **f** G6PD normal. Left panel: **a**, **c**, **e** G6PD activity was determined by the spectrophotometric assay, and right panel: b, d, f by the automated UV enzymatic method. Red circles, yellow triangles, and green squares represent G6PD-deficient, intermediate, and normal newborns, respectively. Red, yellow, and green lines represent linear regression of the percentage of reticulocytes and G6PD-deficient, intermediate, and normal newborns, respectively

In monitoring the reticulocyte counts, reticulocytosis was found in the study population. The mean value of the reticulocyte counts in all newborns with G6PD deficiency was significantly higher than that of normal male and female newborns (*p* < 0.001) (Table 3**)**. The univariate analysis revealed that the reticulocyte counts were significantly positively associated with G6PD deficiency (from both assays) (*p* < 0.001), *G6PD Viangchan*^*G871A*^ (*p* < 0.001), gender (*p* < 0.001), neonatal anemia (Hb < 15 g/dL) (*p* < 0.001), ABO incompatibility (*p* = 0.006), and postpartum age (*p* < 0.001) (Table 5). After adjusting for thalassemia and hemoglobinopathies frequently observed in Thailand, the multivariate analysis revealed that the following variables, including G6PD deficiency, *G6PD Viangchan*^*G871A*^, gender, neonatal anemia, ABO incompatibility, and postpartum age, were independently significantly associated with increasing reticulocyte counts (Table 5). On the other hand, a significant positive correlation between G6PD activity (U/g Hb) from both assays and the percentage of reticulocytes was observed in newborns with normal G6PD status (spectrophotometric assay; *r* = 0.239: *p* < 0.001, automated UV enzymatic assay; *r* = 0.327: *p* < 0.001) but not in G6PD-deficient newborns (spectrophotometric assay; *r* = -0.008: *p* = 0.948, automated UV enzymatic assay; *r* = -0.019: *p* = 0.881) or intermediate newborns (spectrophotometric assay; *r* = -0.218: *p* = 0.019, automated UV enzymatic assay; *r* = -0.296: *p* < 0.001) (Fig. 2a-f).

**Table 5:** Univariate and multivariate linear regression analyses of G6PD status (normal/intermediate/deficient), *G6PD Viangchan*^*G871A*^*/G6PD wild type,* and reticulocyte levels adjusted by gender, hemoglobin levels, postpartum age (day), ABO incompatibility, thalassemia, and hemoglobinopathies

Analysis type	Variable	Reticulocyte counts			
		B*	**SE**	**Beta**	***p*-value**
**Univariate**	G6PD status (Spectrophotometric Assay) (Normal/Intermediate/Deficiency)	0.443	0.076	0.180	** < 0.001**
	G6PD status (Automated UV Enzymatic Assay) (Normal/Intermediate/Deficiency)	0.319	0.073	0.136	** < 0.001**
	*G6PD* Genotype (Wildtype/Mutations)	0.807	0.126	0.197	** < 0.001**
	*G6PD* Genotype (Wildtype/ *G6PD *^*ViangchanG871A*^ / Others)	0.637	0.092	0.213	** < 0.001**
	Gender (Male/Female)	0.398	0.084	0.147	** < 0.001**
	Hemoglobin (≥ 15/ < 15 g/dL)	1.286	0.132	0.292	** < 0.001**
	Thalassemia and hemoglobinopathies	-0.158	0.209	-0.024	0.450
	ABO incompatibility	1.956	0.498	0.162	** < 0.001**
	Postpartum age (day)	-0.314	0.053	-0.184	** < 0.001**
**Multivariate**	**G6PD status** **(Automated UV Enzymatic Assay) (Deficiency/Intermediate/Normal)**	0.316	0.093	0.129	**0.001**
	Gender (Male/Female)	0.470	0.114	0.157	** < 0.001**
	Hemoglobin (≥ 15/ < 15 g/dL)	1.303	0.171	0.292	** < 0.001**
	ABO incompatibility (No/Yes)	1.225	0.466	0.102	**0.009**
	Postpartum age (day)	-0.339	0.064	-0.203	** < 0.001**
	Constant	4.619	0.286		** < 0.001**
**Multivariate**	***G6PD* Genotype** **(Wildtype/Mutations)**	0.630	0.165	0.144	** < 0.001**
	Gender (Male/Female)	0.438	0.114	0.146	** < 0.001**
	Hemoglobin (≥ 15/ < 15 g/dL)	1.264	0.171	0.283	** < 0.001**
	ABO incompatibility (No/Yes)	1.270	0.465	0.105	**0.007**
	Postpartum age (day)	-0.332	0.064	-0.199	** < 0.001**
	Constant	4.984	0.254		** < 0.001**

### Performance of the automated UV enzymatic assay for G6PD enzyme activity detection in newborns

**Fig. 3:**
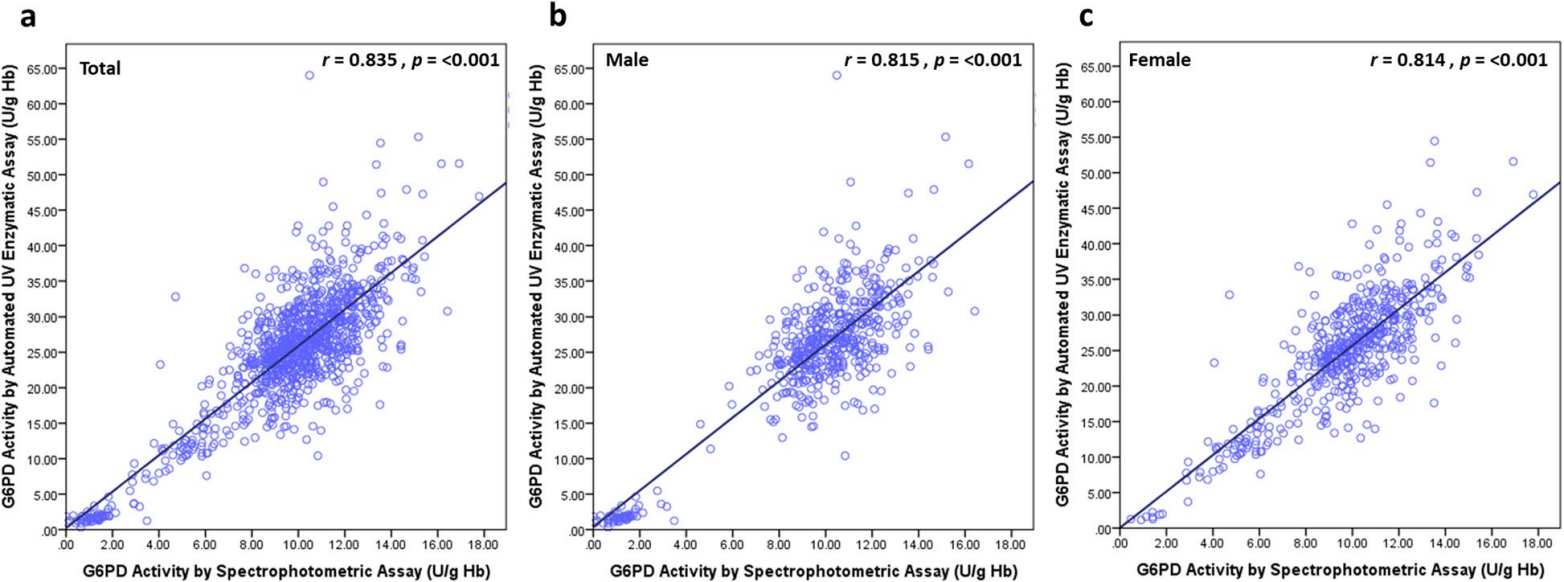
Correlation of G6PD activities measured by the spectrophotometric and automated assays with Pearson correlation

**Fig. 4:**
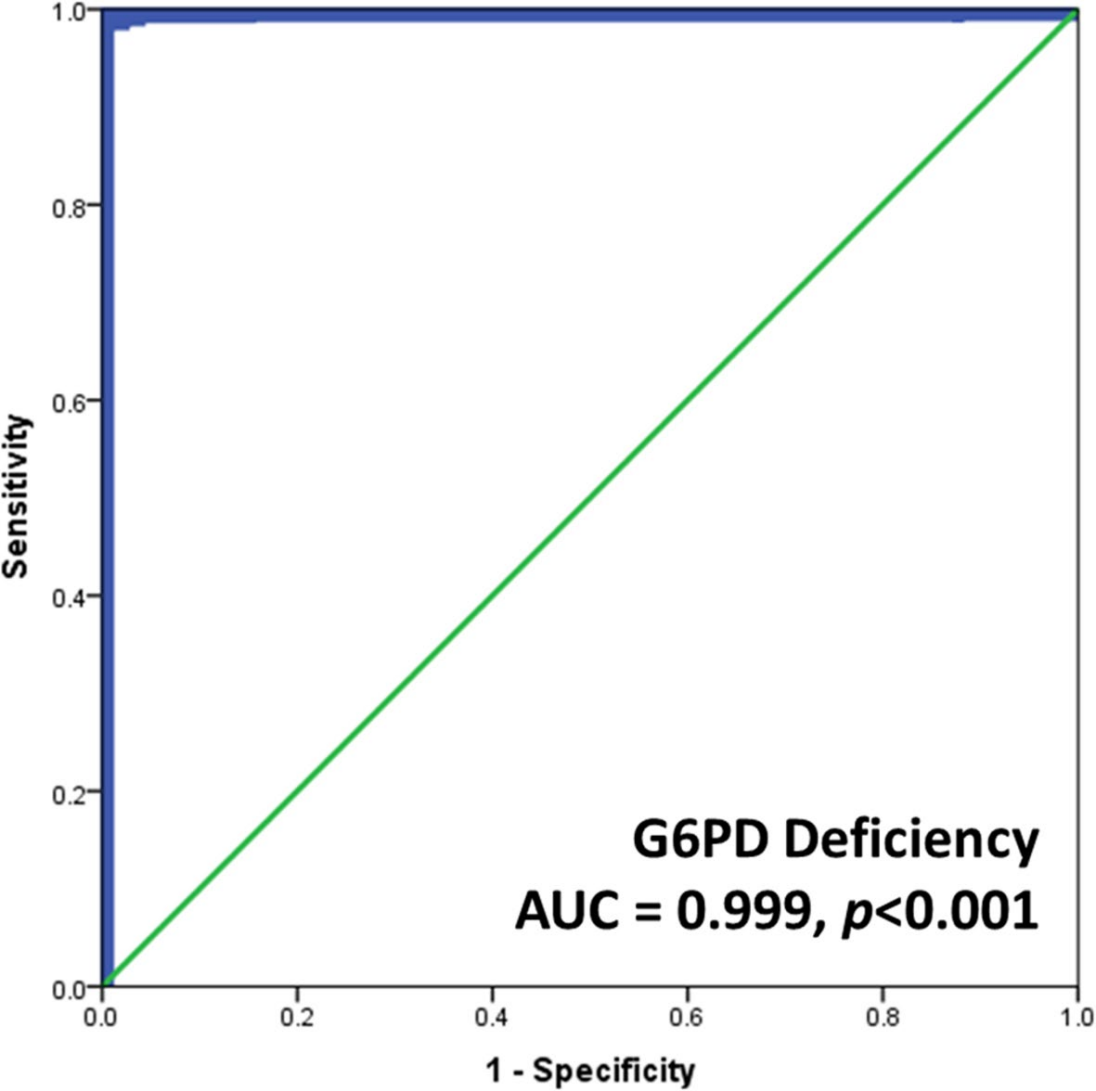
Receiver operating characteristic (ROC) analysis of the automated UV enzymatic method in detecting G6PD-deficient newborns

The median ± IQR of G6PD activity values obtained from the automated UV enzymatic assay were almost three times higher than those obtained from the standard spectrophotometric assay. Nevertheless, G6PD activity levels as assessed by the automated UV enzymatic assay were highly correlated with those of the spectrophotometric assay (*r* = 0.835, *p* < 0.001) (Fig. 3a-c). The proportion of newborn subjects with different G6PD statuses determined by spectrophotometric and automated UV enzymatic assays is illustrated in Table 2. The level of agreement (Cohen’s Kappa) in detecting G6PD status in newborns between the two tests was 0.651. (Kappa’s SE = 0.043 95% CI: 0.566 to 0.735), indicating substantial agreement. The area under the receiver operating characteristic (ROC) curve (AUC) for detecting G6PD-deficient newborns between the spectrophotometric and automated UV enzymatic assays was 0.999 (95% CI: 0.997 to 1.000) (*p* < 0.001) (Fig. 4). The sensitivity, specificity, positive predictive value (PPV), negative predictive value (NPV), positive likelihood ratio, negative likelihood ratio, and accuracy with 95% CI of the automated UV enzymatic method in detecting G6PD-deficient and G6PD intermediate newborns are shown in Table 6.

**Table 6:** Performance of the automated UV enzymatic method and FST in detecting G6PD deficiency and G6PD intermediate newborns

G6PD status	G6PD activity cut-off value (U/g Hb)	Sensitivity (%) (95%CI)	Specificity (%) (95%CI)	PPV (%) (95% CI)	NPV (%) (95% CI)	Positive Likelihood Ratio (95% CI)	Negative Likelihood Ratio (95% CI)	Accuracy (%) (95% CI)
*FST*								
Deficiency	-	87.1 (76.2–94.3)	100.0 (99.6–100.0)	100.0	99.2 (98.4–99.6)	-	0.1 (0.1–0.3)	99.2 (98.5–99.7)
*Automated UV enzymatic method*								
Deficiency	< 7.8	98.4 (91.3–100.0)	99.5 (98.8–99.8)	92.4 (83.6–96.7)	99.9 (99.3–100.0)	187.5 (78.2–449.8)	0.02 (0.0–0.1)	99.4 (98.7–99.8)
Intermediate	7.8–20.8	69.0 (59.7–77.2)	91.3 (89.3–93.1)	50.6 (44.5–56.7)	95.8 (94.6–96.8)	8.0 (6.2–10.2)	0.3 (0.3–0.5)	88.8 (86.7–90.7)

Based on a 10% G6PD deficiency prevalence in our male subjects reported previously (Domingo GJ. *et al.* 2019), the proportion of G6PD deficiency in females detected by both assays at 30%, 40%, 60% and 80% threshold G6PD activity is shown in Table 7.

**Table 7:** The prevalence of G6PD deficiency in male and female newborns, assuming different thresholds for deficiency. The prevalence was calculated based on G6PD activity measured by spectrophotometric and automated UV enzymatic assays

**Male G6PD deficiency prevalence**		**Threshold G6PD activity expressed as percent of normal**																		
**At 10%**		30%				40%				60%				70%				80%		
		M	F	T		M	F	T		M	F	T		M	F	T		M	F	T
**Domingo GJ. *et al.* (2019)**	No. def	500	124	624		500	237	737		517	656	1173		595	968	1563		864	1313	2177
	% Def	80	**20**	100		68	**32**	100		44	**56**	100		38	**62**	100		40	**60**	100
	% Pop	10.0	**2.5**	6.2		10.0	**4.7**	7.4		10.3	**13.1**	11.7		11.9	**19.4**	15.6		17.3	**26.3**	21.8
***G6PD activity method***	No	502	513	1015		502	513	1015		502	513	1015		502	513	1015		502	513	1015
**Spectrophotometric assay (n)**	No. def	50	12	62		52	17	69		56	57	113		58	78	136		79	99	178
	% Def	80.6	**19.4**	100.0		75.4	**24.6**	100.0		49.6	**50.4**	100.0		42.6	**57.4**	100.0		44.4	**55.6**	100.0
	% Pop	10.0	**2.3**	6.1		10.4	**3.3**	6.8		11.2	**11.1**	11.1		11.6	**15.2**	13.4		15.7	**19.3**	17.5
**Automated UV enzymatic assay (n)**	No. def	52	14	66		52	23	75		61	74	135		75	97	172		99	125	224
	% Def	78.8	**21.2**	100.0		69.3	**30.7**	100.0		45.2	**54.8**	100.0		43.6	**56.4**	100.0		44.2	**55.8**	100.0
	% Pop	10.4	**2.7**	6.5		10.4	**4.5**	7.4		12.2	**14.4**	13.3		14.9	**18.9**	16.9		19.7	**24.4**	22.1

## Discussion

G6PD deficiency is an important risk factor for severe hyperbilirubinemia and bilirubin neurotoxicity in newborns [6]. The prevalence of G6PD deficiency differs between geographic regions and ethnic groups. For example, the prevalence of G6PD-deficient males was 7.3% in Thailand, 8.1% in Lao PDR, 15.8% in Myanmar, 18.8% in Cambodia, and 8.9% in Vietnam [23]. In this study, our spectrophotometric analysis recommended by the WHO as a gold standard method revealed that the frequency of G6PD deficiency in Thai male newborns was 10% and 2.3% for males and females, respectively. The incidence in male newborns was in agreement with that reported by Bancone *et al.* [23], but the incidence in female newborns was lower than that reported by Nuchprayoon *et al.* [6]. However, G6PD intermediate was more prevalent in female newborns than in male newborns. The possible reasons for the differences might be random X inactivation of female heterozygotes. According to the molecular analysis of the *G6PD* genotype, the most prevalent mutation in the study population was *G6PD Viangchan*^*G871A*^ (MAF = 0.066). This finding was consistent with other reports [9, 11]. The hemizygous males and homozygous females for *G6PD Viangchan*^*G871A*^ had severe to moderate deficiency, whereas the *G6PD Viangchan*^*G871A*^ heterozygous female phenotype varied from moderate deficiency to normal. Additionally, frequent mutations in Southeast Asia and China, including *G6PD Canton*^*G1376T*^*, G6PD Kaiping*^*G1388A*^*, G6PD Mahidol*^*G487A*^*, G6PD Songklanaklarind*^*T196A*^*, G6PD Chinese-4*^*G392T*^*, G6PD Union*^*C1360T*^*, G6PD Chinese-5*^*C1024T*^*, G6PD Valladolid*^*C406T*^*,* and *G6PD Aures*^*T143C*^ were found in the study population. Both homozygous females carrying *G6PD Kaiping*^*G1388A*^ and *G6PD Chinese-5*^*C1024T*^ had deficient phenotypes, whereas heterozygous mutants were found in intermediate subjects. As the inheritance of *G6PD* is X-linked, homozygous females and hemizygous males fully express the deficiency phenotype, whereas heterozygous females present partial expression as a result of random X inactivation.

Screening for G6PD deficiency in newborns has been encouraged in many countries with high incidence to prevent jaundice and possible consequences and to raise parental awareness about their children’s condition. It has been suggested that the presence of a large number of reticulocytes may interfere with the diagnosis of G6PD deficiency since reticulocytes have more G6PD enzyme activity than mature erythrocytes [4]. They are released into the bloodstream in response to G6PD deficiency-induced hemolytic anemia [24]. Moreover, the reticulocyte count is higher at birth than at any other point in a healthy life [25]. This led us to investigate the effect of reticulocytosis on G6PD activity in Thai newborns using the automated platform. We found that the mean reticulocyte count in this study population was 4.87 ± 1.36% (range 0.98–12.38), which is higher than that in adults (generally 0.5%-2.5%). It is interesting that the reticulocyte counts in newborns with G6PD deficiency were significantly higher than those in normal newborns in both males and females. After adjusting for thalassemia and hemoglobinopathies commonly observed in this region, we found that G6PD deficiency, *G6PD Viangchan*^*G871A*^, female gender, neonatal anemia (Hb < 15 g/dL), ABO incompatibility, and postpartum age were independent factors promoting reticulocytosis in newborns. These novel findings indicate that increased reticulocyte status in newborns with G6PD deficiency and intermediate is a result of the compensatory effect of reduced enzymatic activity. However, our results demonstrated that the levels of G6PD activity in normal newborns were highly positively correlated with the percentage of reticulocytes obtained from both assays. Even though the results supported an increase in reticulocytes in G6PD deficiency compared to G6PD normal, there was no association between G6PD activity levels and the percentage of reticulocytes in newborns with G6PD deficiency. This finding agreed with a previous report [26] that high reticulocytosis in neonatal blood samples did not cause a false negative diagnosis for G6PD deficiency. G6PD deficiency is a genetic disorder caused by mutation in the *G6PD* gene affecting G6PD deficiency in all cell types, including reticulocytes. Although there are numerous reticulocytes in the circulation during the hemolytic crisis as a result of G6PD deficiency, their G6PD activity cannot increase the total blood G6PD activity to the normal level. Hence, elevated levels of G6PD activity in reticulocytes only interfere with the detection of G6PD activity in normal blood samples. In newborns with intermediate levels of G6PD, the levels of G6PD activity were highly negatively correlated with the percentage of reticulocytes. This might result from the presence of a mixed population of G6PD deficiency and normal reticulocytes as a result of heterozygosity.

Since the number of reticulocytes in normal samples affected the measurement of G6PD activity, the efficacy of the automated assay was assessed. Our results revealed that the frequencies of G6PD-deficient males and females obtained qualitatively by using the FST and quantitatively by using the automated UV enzymatic method were not different from those obtained by using the standard spectrophotometric method, indicating the reliability of the automated method. Although the FST method is a simple and rapid qualitative method for screening G6PD deficiency, as recommended by the International Committee for Standardization in Hematology (ICSH) [23, 27], it does not well serve the purpose of separating G6PD intermediate from G6PD normal. The quantitative automated UV enzymatic method is a new robotic spectrophotometric system that is capable of quantifying G6PD activity in 50 blood samples simultaneously. The bimodal and trimodal distribution patterns of G6PD activity values observed in this study population coincided with the characteristics of hemizygous G6PD deficiency and normal males and heterozygous and homozygous G6PD deficiency and homozygous normal females, respectively. Based on previous recommendations, this study used 30% and 80% of normal median activity as cut-off points for G6PD deficiency and intermediate, respectively [15]. In our study, the detection of G6PD deficiency in females at 30% of normal activity by the automated UV enzymatic assay was close to the estimated frequency of G6PD-deficient females reported previously by Domingo GJ. *et al. *2019 [22]. The results from ROC analysis revealed that the optimal cut-off value of the automated UV enzymatic method for G6PD deficiency in newborns was less than 7.8 U/g Hb, which was comparable to the value reported previously [26], and for those with intermediate levels of G6PD activity, it was 7.8 to 20.8 U/g Hb. The diagnostic performance of the automated UV enzymatic method was practically faultless for G6PD deficiency, while the effectiveness of intermediate screening was reasonable.

Our results also revealed that the normal reference values of males and females determined by the automated UV enzymatic method were not significantly different. We also found that the reference values of the automated UV enzymatic method for the identification of deficiency and intermediates among newborns were higher than those of adults reported previously [11]. This finding is in agreement with previous reports that G6PD activity in normal newborn blood is higher than that in adult blood [28, 29]. In contrast to the spectrophotometric assay, our reference values for G6PD-deficient and intermediate newborns were comparable to those of adults [11]. Although the G6PD activity values from both methods showed a strongly positive correlation (Fig. 3a-c), we are aware that G6PD activity values obtained from the automated UV enzymatic assay are excessive by a factor of almost three. We plan to introduce corrections in our automated assay until the results are in line with those that have been long established. Both techniques are based on the same principle in measurement the absorbance of NAPDH production at 340 nm and 37 °C. The high values of G6PD activities from the automated UV enzymatic assay may be explained by two reasons: (i) the differences in sample preparation for each assay and (ii) Hb measurement for normalization. The packed red cells were used for the automated UV enzymatic assay, whereas the whole blood was used for the spectrophotometric assay. The packed red cell samples were prepared by centrifugation and removal of plasma and the buffy coat containing the white blood cells. The enrichment of reticulocytes in packed red cell samples may result in high G6PD activity values, compared to whole blood samples in which all cellular contents including reticulocytes were diluted with blood plasma. In this study, Hb values used to normalize the G6PD activity values from both assays were obtained from CBC analysis of whole blood. Hence, we propose that Hb values suitable for normalization of G6PD activity from the automated UV enzymatic method should be derived from the packed red cell hemolysate, rather than from the whole blood.

In summary, reticulocytosis, commonly observed in newborn G6PD deficiency, did not interfere with the diagnosis of G6PD deficiency and intermediate. However, G6PD activity in the normal group was positively correlated with the percentage of reticulocyte count. This affects the activity of G6PD as measured by the automated UV enzymatic method. Nevertheless, the robotic quantitative method using reticulocyte-enriched packed red cells shows a strong diagnostic capability to identify G6PD deficiency and intermediates with high cutoff values. A quantitative spectrophotometric assay is suitable to quantify newborn G6PD activity and detect G6PD deficiency in the prevention of hemolytic anemia of G6PD deficiency.

## Data Availability

All data generated or analyzed during this study are included in this published article.

## Abbreviations

G6PD: Glucose 6-phosphate dehydrogenase
FST: Fluorescent spot test
ROC: Receiver operating characteristic
PPV: Positive predictive value
NPV: Negative predictive value
HMP: Hexose monophosphate shunt
NADPH: Nicotinamide adenine dinucleotide phosphate
AHA: Acute hemolytic anemia
AMM: Adjusted male median
Hb: Hemoglobin
IQR: Interquartile range
PKU: Phenylketonuria
AUC: The area under the receiver operating characteristic curve
HDFN: Haemolytic disease of the foetus and newborn
MAF: Minor allele frequency
ICSH: International Committee for Standardization in Hematology
WHO: World Health Organization
EDTA: Etheylenediaminetetraacetic acid
